# A Qualitative Study to Explore the Employment Experiences and Perspectives of Women Living with Multiple Sclerosis in the UK

**DOI:** 10.1007/s10672-022-09413-6

**Published:** 2022-06-22

**Authors:** Cathie Railton, Laura Jefferson, Jo Taylor

**Affiliations:** Department of Health Sciences, University of York, York, England

**Keywords:** Multiple sclerosis, Women, Employment, Qualitative, Disability

## Abstract

Employment is a key determinant of health, yet up to 80% of individuals with multiple sclerosis stop work within 15 years of diagnosis. The unpredictable nature of MS, both on a daily basis, and longer term means that maintaining employment can be difficult. Multiple sclerosis affects women disproportionately (> 60% of cases) and they often experience greater workplace challenges, yet few studies explore this. This study aimed to deepen understanding of the employment experiences and perspectives of women with multiple sclerosis to help inform future policy developments and care. A descriptive phenomenological approach was used involving thematic analysis from fourteen in-depth semi-structured interviews (data collected 2020). The inclusion criteria were adult women diagnosed with multiple sclerosis living in the UK who have current or previous experiences of employment. Seven themes were identified: Multiple sclerosis symptoms, workplace support, adjustments in the workplace, prioritisation of employment, making compromises, time and informal networks. These themes offer a descriptive account of the participant’s experiences of work, how they experience their multiple sclerosis in relation to employment and some of the constraints and enablers to work. The findings highlight the importance of both individual and broader socio-environmental factors to successful employment outcomes. Tailored community support for these women, such as that provided by nurses, was considered central. There is a need for better collaboration at a policy level between government departments and for more research into women with chronic conditions, to further explore the relationship between different variables.

## Background

Multiple sclerosis (MS) is a lifelong chronic neurological condition, with an aetiology that is still mostly unknown (MS Trust, 2018). Symptoms vary considerably and can include difficulty with walking, balance and co-ordination and cognitive issues (NHS Choices, 2019). Depending on the type of MS, symptoms may come and go in phases or get steadily worse over time. The unpredictable nature of MS, both on a daily basis, and longer term means that maintaining employment can be difficult. Symptoms are often invisible and can fluctuate daily, making it challenging for individuals to undertake tasks confusing to others with less understanding of the condition.

MS affects 2.5 m people globally. The proportion of women with MS is increasing; affecting between two to three times as many women as men (MS Trust, 2020). In the UK, Public Health England (2020) state that there are 131,720 people living with MS, with 105,800 (190 per 100,000) of those in England. Most new diagnoses (93%) amongst women occur in the 30 to 34 years and 40 to 44 years age groups (Public Health England, 2020); often an important time for making decisions about work and family.

The relationship between employment and health has been well documented, for example Dahlgren and Whitehead's (2007) social model of health outlined the impact of social determinants, such as living and working conditions, on health. The long-term unemployed have a lower life expectancy and worse health than those in work (Public Health England, 2017); as is also the case for people with MS. A recent review found individuals with MS working full- or part-time had better general health and well-being, including higher quality of life and lower symptoms of depression (Dorstyn et al., 2017).

Despite legislation to protect individuals from discrimination at work on the grounds of personal characteristics such as sex or disability (HM Government Equality Act, 2010) and women’s increasing participation in employment, disparities in working patterns still exist by sex and disability. Female employment rates are 8% below those of men and women continue to earn less than men (Office for National Statistics, 2020). While this can be partly attributed to them carrying out more part time roles, women also tend to work in lower paid occupations and struggle to reach higher levels in organisations. Furthermore, in the UK only 53.2% of disabled people are in work compared to 81.8% of non-disabled people (Office for National Statistics, 2019).

The role of health and social inequalities has been the focus of UK policy for some time. The 2010 Marmot Review (Marmot et al., 2010) and the 2017 White Paper ‘Improving Lives: The future of work, health and disability’ (Department for Work and Pensions and Department of Health, 2017) placed emphasis on enabling disadvantaged groups in the workplace and transforming employment opportunities for people with disabilities. However, a recent review found that whilst UK employment rates had improved, there was an increase in poor quality employment such as zero hours contracts, insecure employment and sex and disability inequalities continue (Marmot et al., 2020).

There are few studies that explore MS and employment in the UK. The All Party Parliamentary Group (APPG) for MS (2017) consulted over 1,500 people with MS and health professionals to identify barriers to employment and made recommendations for government, employers and service providers. Survey respondents raised fatigue as the main symptom of MS which limited the types of work, but more detrimental were the lack of support services and unhelpful employer attitudes. Only 55% of people with MS were working and up to 80% of people with MS retire within 15 years of diagnosis (All-Party Parliamentary Group for MS, 2017). Whilst informative, fundamentally there was no analysis around sex differences. A recent systematic review of factors influencing workforce participant for people with MS (Goodwin et al., 2021) also found studies tend not to explore wider social and cultural factors affecting employment, such as gender.

A small number of studies focus specifically on women with MS and employment, however these are from US and Canadian settings (Arnett & Strober, 2016 and Jongbloed, 1996). Jongbloed surveyed over 800 women and carried out in depth interviews of 54 women. Supportive work and home environments were the main factors enabling women to continue working and that their employment decisions were determined by the social context in which they function. Arnett and Strober (2016) explored the role demographics and disease variables at play in employment of women. 68 women completed assessments measuring cognition, disease symptoms, psychological functioning, coping and stress, with social support and coping style differing in those with MS in employment and those who left work. Furthermore, those who were still working generally had a more positive view of work, seeing the benefits of being employed for their overall health and wellbeing (Arnett and Strober 2016).

These studies highlight the different factors that influence individuals with MS (and more specifically women) and their relationship with employment. However, whilst informative, they are limited to countries outside the UK where policies and cultures differ and their health, employment, disability law and welfare systems vary. In their paper ‘Disability and employment: A comparative critique of UK legislation’, Goss et al. (Goss et al., 2011) discuss the differences between UK and US disability legislation and say that in the US there is a greater focus on anti-discrimination measures. They say that the UK’s disability definitions are more restrictive, however, both countries do specifically recognise MS as a condition that can qualify for disability benefits.

To our knowledge, there is no UK study exploring the experiences and perspectives of women living with MS in the UK regarding employment. This study aims to address this gap, gaining a rich, in-depth understanding of the experiences and perspectives of women with MS regarding employment in the context of living and working in the UK.

Study objectives included:

- To understand how women with MS experience and make sense of paid employment in their daily lives.
- To identify factors influencing women’s decisions to work following a diagnosis of MS (including constraints and enablers).
- To identify the support and information needs of women with MS regarding paid employment.

## Methods

### Methodology

This study uses descriptive phenomenology to explore the employment experiences of women with MS. Writing in the first decade of the twentieth century, descriptive phenomenology was founded as a philosophical movement by Edmund Husserl. Husserl argued that ‘objects *in the world were not passively understood but were actively constituted through consciousness and subjective experience. To understand the “essence” of phenomena, one had to understand how the “lifeworld” was directly experienced’* (Green and Thorogood, 2018, p.42). This type of phenomenology enabled employment to be explored through the experiences and perspectives of women living with MS, and identify key factors affecting their employment desires, experiences and futures. Descriptive phenomenology also uses ‘bracketing’ as a key process, whereby the researchers ‘bracket off’ their preconceived ideas and beliefs about the phenomena under study and constantly review them throughout the research process. This was particularly important because the primary researcher is a woman living with MS who is employed, so participants’ lived experiences were considered while attempting to limit researchers’ own assumptions during the analysis.

### Sampling

Adult women (aged 18 and over) and diagnosed with MS living in the UK were included if they had current or previous experiences of paid employment.

Based on existing literature of factors influencing employment choices and experiences (Arnett & Strober, 2016 and Jongbloed, 1996), a criterion sampling strategy was used with the aim of achieving a sample of women who, while sharing a diagnosis of MS and experiences of employment, varied across the following key characteristics: age, type of employment, education level and length of time since diagnosed with MS. Whilst there is no agreed set sample size for phenomenological studies, Creswell (1998) recommends between 5–25 and Morse (1994) suggests at least 6 before reaching saturation.

### Recruitment

Participants were identified through the national MS Society and through local networks. Nationally, a flyer was sent to women on their general mailing list (several thousand), those who attended support groups and also to women who regularly fundraise for the organisation. Different routes were used to try and ensure a diverse sample were reached, i.e. those working and not working, those who are newly diagnosed and diagnosed for several years and younger and older women. Local MS Society groups in Yorkshire were also asked to publicise the work through their social media accounts.

17 women expressed an interest in the study. They made initial contact by text, phone or email, following which, the researcher responded the same way to briefly explain discuss the aims of the study as well as confidentiality and consent. Three women did not respond at this stage, with the remaining 14 consenting to interview having read the study participant information sheet.

### Patient and Public Involvement

The study was discussed with the MS Society (a national charity that funds research and campaigns for people living with MS) who facilitated the involvement of individuals living with MS in the focus of the work. The charity has an ‘Experts by Opinion Group’ which was used to help shape the study through advising on the interview topic guide and commented on the initial findings.

### Data Collection

In-depth semi-structured interviews were used to collect data, a method commonly used in phenomenological studies which seek to collect rich data about individual experiences (Creswell, 1998). Interviews took place during the early stages of the COVID-19 pandemic, so all were conducted by telephone.

Informed by the existing literature, and with input from the MS Society Experts by Opinion Group lead, a topic guide was developed. The topic guide was piloted with the MS Society Group lead prior to the interviews taking place and the final iteration had five key headings; basic information (e.g. demographics), their MS, education and employment, support networks and other information (see appendix Table 2).

Interviews took place over a 4-week period between February and March 2020 and lasted on average for 52 min. As well as making written notes, an encrypted digital audio recorder was used to record interviews. The interviews were then transcribed and uploaded to NVIVO 12 (QSR International Pty Ltd 2018) for analysis, they were not returned to participants for comments. The primary researcher conducted all interviews, there were no repeat interviews carried out and participants were not asked for feedback on the findings (Appendix Table 3).

### Analysis

An iterative approach was employed to classify and organise the data from the interviews (see appendix 4). Thematic analysis was used as this allows the researcher to explore the experiences of participants and the meanings they attach to these experiences (Sundler et al., 2019). As the research adopted a descriptive phenomenological approach focussing on the lived experience of participants, an inductive approach to thematic analysis was employed based on the six phases outlined by Braun and Clarke (2006, p.35), (see Appendix Table 4).

### Reflexivity

All three researchers are professional women in employment. The primary researcher is a postgraduate student in public health and also has personal experience as a woman diagnosed with MS for one year before conducting the study. Reflexivity therefore formed an important component of the study. The primary researcher was able to fully understand the condition and gain deeper insight through her own lived experience, however, as a team, we were able to constructively challenge each other’s beliefs and assumptions which helped to minimise subconscious bias.

### Ethics Approval

Ethical approval (HSRGC/2019/364/B) was gained through the University of York’s Health Sciences Research Governance Committee.

## Results

The characteristics of the 14 women who took part are displayed in Table 1. All participants were living with chronic MS and length of time since being diagnosed ranged from less than 5 years up to 20 years. All the women were British, and all but one described themselves as white. Most participants were from Yorkshire, while others were from the North West, Midlands, the South of England and Scotland. All but three of the women had children; around half of whom were still dependant and living at home. There was an even split between women who were still working and those not working. The type of work participants carried out varied; most of them (9/14) had undertaken a degree and went on to professional careers in teaching and nursing, others continued their education up to 18 years old and worked in hospitality, retail and administration.

**Table 1:** Characteristics of participants

Participant ID		Age group	Children	Living with	Years since diagnosed	Employed	Educational level	Occupational group
1,20 > ,nw		60–69 yrs	Yes	Children	> 20 yrs	No	Masters	Nursing/midwifery
2,20 > ,nw		50–59 yrs	No	Alone	> 20 yrs	No	College until 18 yrs	Administration
3,10 > ,w		40–49 yrs	Yes	Partner & children	11–20 yrs	Yes	College until 18 yrs	Hospitality
4, > 20,w		60–69 yrs	Yes	Partner	> 20 yrs	Yes	Degree	Teaching
5,5 > ,w		50–59 yrs	Yes	Partner	5–10 yrs	Yes	Not known	Local government
6,20 > ,nw		40–49 yrs	Yes	Partner & children	> 20 yrs	No	Degree	Communications
7,20 > ,w		60–69 yrs	Yes	Partner	> 20 yrs	Yes	College until 18 yrs	Retail
8,20 > ,nw	70 yrs. +		No	Alone	> 20 yrs	No	Degree	Teaching
9,10 > ,nw	50–59 yrs		Yes	Partner & children	11–20 yrs	No	Degree	Teaching
10,10 > ,w	60–69 yrs		Yes	Alone	11–20 yrs	Yes	College until 18 yrs	Retail
11, < 5,w	50–59 yrs		Yes	Partner & children	< 5 yrs	Yes	Degree	Nursing/midwifery
12, < 5,w	20–29 yrs		No	Partner	< 5 yrs	Yes	Degree	Registered veterinary nurse
13, > 20,nw	40–49 yrs		Yes	Children	> 20 yrs	No	Degree	Administration
14 > 20,nw	50–59 yrs		Yes	Partner	> 20 yrs	No	Degree	Nursing/midwifery

Participants were given an identification number with abbreviations providing information on key characteristics including participant number, years since diagnosed, working status (w: working, nw: not working).

### Thematic Findings

The interviews provided rich data about women with MS and their experiences regarding employment and some of the constraints and enablers to work, categorised under seven themes. These provide insight and understanding about the concealed meanings in relation to their employment experiences, linking the women’s experiences together.

#### MS Symptoms

All women reported their symptoms as a barrier to them being able to work. Fatigue was the most common symptom referred to, but others included eye sight, cognitive impairment, a loss of sensation in their limbs, poor balance and pain. The type of work, lack of workplace support, physical environment (e.g. mobility gripping aids in place) and being able to get to work confidently were also referred to as barriers.

*There are certain jobs where you would struggle like retail.*

Interview 5,5>,w

*Sometimes it’s quite hard. Obviously, it depends how I’m feeling. I can get really tired and whenever I finish at 2pm I literally just fall asleep. I plan to come home and do things, but I end up falling asleep all afternoon. So, it is still quite tiring doing the work itself and I do feel like when I'm at work sometimes I find it harder to do activities in the day.*

Interview 12, <5,w

The way the women experienced their MS impacted on their work and work choices. Symptoms varied greatly and almost all participants reported a delay in diagnosis and some were repeatedly misdiagnosed by doctors. In addition to impacting directly on their work decisions, it also effected confidence and relationships with others. For one woman, this had contributed to her attempting suicide. Some older women with MS for over 20 years described that their diagnosis had been kept from them by medical professionals due to perceived wisdom at the time.

*‘I just assumed that was that was just me, that's how I was and also depression came into it and 2 suicide attempts came into it—just through not knowing what was wrong with me.’*

Interview 8,20 > ,nw.

Many of the women referred to the unpredictability of their MS, which made being able to work a challenge, whether this be due to a relapse which could mean they were unable to work for several months or because their symptoms flared up without any warning.

#### Workplace Support

Participants described the period from experiencing the symptoms to getting a diagnosis as challenging as they knew they were unwell but didn’t feel they could speak to anyone about it until they were diagnosed. Even once diagnosed, not all the women felt able to disclose their condition at work until they had come to terms with it themselves and they described being concerned about the reactions of colleagues. The most common concern expressed by participants was that their employer and colleagues would think they were no longer competent in doing their job:

*I won't tell everybody at work because you don't want people to think you can’t do the work…… I always worry that someone’s going to say I’m not capable of doing my job and take me down the capabilities route.*

Interview 5,5 > ,w

None of the women described proactive and continuous support from their employers and there was frustration expressed by those women who worked for larger organisations with multiple management layers about the lack of co-ordination and understanding between different managers. The level of support and understanding they received from their management often relied on the individual manager, rather than the organisation’s policies and approach to disability:

*My new line manager checks in with me every day so the issues that I was having with my old one is I found out like she was saying that she didn't believe that I was an unwell or going to my appointments, then when I requested the reasonable adjustments like working from home, she just said no straight away. So, it was kind of borderline discrimination.*

Interview 12,<5,w

At times negative reactions from management and colleagues resulted in women leaving their roles, rather than any physical impediment. These negative experiences at work had a long-lasting impact on motivation to work for some women. After having a particularly challenging time with her employer, one woman spoke about seeking legal advice and union support in order to be given reasonable adjustments at work. Ultimately, she felt she had to leave the role:

*I was like I can't fight a massive company on my own I just can't do it and I was getting over a relapse as it was, but it literally got me when I was down. And I was like I can't fight this on my own so essentially, I handed my notice in and left because I had no other option.*

Interview 3,10>,w

#### Adjustments in the Workplace

Not all participants reported employers making reasonable adjustments, as the responses discussed in the previous theme would suggest. Where these were supported by employers, this resulted in a more positive employment experience as women felt supported and equipped to carry out their role.

*I've just started with working from home as well, so I think that will help with my energy levels too.*

Interview 12,<5,w

Adjustments that had been made by employers included a change in working patterns (e.g. reduction or change in hours), reduction in shift work, flexibility to work from home, provision of equipment (e.g. chair, software), review of responsibilities and repositioning to an office closer to toilet facilities.

All participants also described strategies they had used to help them remain in work. These included understanding the triggers of their MS symptoms, taking rests if working on their feet regularly, feeling able to ‘open up’ about their MS with colleagues and managing their time around their symptoms. For example, one woman described working on more challenging tasks at her *“best times”:*

*It’s about knowing your best times. So, when I was at work, if I had very important meetings, I made sure they happened in the morning because I know I’m on the ball in the morning.*

Interview 14>20,nw

#### Prioritisation of Employment

Women were asked about their experiences in relation to support they have received not just from the hospital MS services, but also wider health and wraparound support (GP, benefits, job centre etc.). Responses were mixed with none of the participants recalling any conversations about work when diagnosed and only two women reporting support later on. Instead, the focus tended to be around symptom management, aids and adaptations and treatment options rather than other wider but related issues that impact on their health (work, relationships, housing and lifestyle).

Many women spoke very highly of their MS nurse, describing their support as invaluable. This was, however, generally focussed very much on their MS and symptom management rather than wider issues which contribute to their overall health and wellbeing. The capacity of nurses was perceived by some participants as limited, but overall participants believed that wider support should also be made available at the time of diagnosis and on an ongoing basis.

The lack of co-ordination between different services was raised by some participants as an issue that affected their work. For example, one woman explained that the hospital services and health professionals were fragmented, which she felt impacted her ability to remain in regular employment due to time constraints:

*I was having a lot of time off because the problem is it's not a coordinated system so you need this morning off to get the neurologist, then you need another afternoon on Wednesday to go and see the MS nurse and then the physio has to come to your house when you're in on another day - it's all fragmented.*

Interview 1,20 > ,nw

#### Making Compromises

Without exception, all participants reported their MS impacting on their work negatively. For some this had completely changed their employment trajectory, while others described adapting it. They referred to having to make compromises throughout their MS journey in relation to employment because of the barriers to work they experienced and their ability to continue in roles held prior to diagnosis. Some participants described these positively as choices that led to feelings of fulfilment through reprioritising life and their health. For others, compromises were less positive, with some women perceiving limited choices about continuing in their role or in employment at all.

Some women described refusing to let their diagnosis define them and actively pushed themselves. For example, one older participant who could not walk without the use of a mobility aid described how she still teaches yoga:

*I can’t walk really anymore; I walk with a rollator. I have taxis come round to me and pick me up for my yoga.*

(Interview 4,>20,w).

For others, the wider effects of mental health and relationship impacts had led to compromises in their employment choices. One of the women reported suffering considerably with her mental health (undiagnosed at the time), which prevented her from continuing work:

*If I’d been told when I kept going to the doctor asking why I was so tired, and you know worrying about depression, but nobody ever mentioned MS. If they’d had said it could be MS, let's send you to a neurologist say in the 80s then I would definitely have been able to change my work from full time to part time and carry on that would have suited me no end.*

Interview 8,20 > ,nw.

#### Time

Time was a recurring theme throughout all the interviews. Discussed in relation to the process of denial and acceptance about their condition, as well as changes in MS symptoms and understanding of symptoms over time. It also related to changes in support available over time from wider services and changes in employment laws.

Participants described how these changes impacted on their experiences of employment and decisions about work. They described certain points in time as being prominent and critical in terms of their support needs and ability to make informed decisions around employment, i.e. at the point of diagnosis and in the short term after this until they had learned to live with the condition better.

All the women were very open to having discussions about work with their MS nurse. They described how their needs changed over time and this impacted on their decisions about employment, suggesting that conversations about work should take place regularly throughout the various stages of disease progression.

*I think that when I was diagnosed and when I was working there was nothing that could be put in place. I think it changed very much in recent years it is expected that companies do put those things in place but that's only a very recent thing for people with disabilities and I’ve not really worked in that time.*

Interview 13, > 20,nw.

Reflection was particularly evident amongst the older women as their symptoms had often progressed over time and they talked about the changes in support available which, if they had received that at the time they were diagnosed, would have changed their employment choices. Participants expressed a sense of loss over not having access to the support which could have had changed their decisions around employment.

#### Informal Support Networks

The importance of support networks was apparent throughout the analysis. Women experienced less difficulties in employment when describing access to resources such as support from their partner/family support and MS team, supportive workplaces and having other interests (hobbies/pastimes). The women who described less support were more likely to stop working and described making considerable sacrifices. This was particularly difficult for the women who have been diagnosed for longer as many improvements have since been made in terms of support services and employment law.

Women also spoke of the need to be open and honest about their condition and the use of humour to cope with challenges faced on a daily basis.

*They're (her children) not bothered they have to push their Mother in a wheelchair and my husbands not bothered. He’s very much like you've got to get out and he’s absolutely right. There’s lots of comedy about it, we all get hysterical.*

Interview 11,<5, w

Support from the MS community was discussed in relation to social media groups, knowing other people with MS or going to MS support groups. Access was difficult for women still working as many groups were held during the day. Some also described hesistancy in attending support groups because they didn’t feel they could relate to other attendees that may no longer be in employment. Some women spoke about how their perceptions changed over time and, whilst early on in their MS journey they didn’t see the benefit of such groups, they later came to value the support. This view was generally expressed by those no longer working.

*I never really joined until I left work, but I would have joined if I’d known earlier how supportive they can be.*

Interview 14>20,nw

## Discussion

### Key Findings

This study provides important insights about the complex and changeable interplay between MS and paid work in women’s lives and the factors that influence their work experiences and choices. The findings showed that while MS symptoms do impact on work, this varied considerably between participants and over time, and depended on the nature of their employment as well as the support they had from employers, colleagues, MS nurses and informal support networks. Age was not found to be a factor in why women stopped working but length of time since diagnosis was, not due to having more advanced symptoms but rather the lack of support received whilst employed. The study highlights how difficult some women found it to disclose their MS diagnosis to their employer, and the importance of the individual work of the women as they negotiated with employers about adjustments and implemented their own strategies to enable them to continue in their role. For some women, the lack of support from employers led to them leaving work and impacted adversely on their health. For others, employment experiences were more positive; however, all of the participants described having to make compromises in their employment choices although the consequences of these varied by participant and over time.

Time was an important concept identified from the data in relation to the way the women’s needs, and their perceptions and understanding of these, changed as their condition fluctuated and progressed, and their personal and working lives evolved. This highlights the need for healthcare staff to integrate conversations about work as a routine part of their practice with MS patients, and for employers to engage routinely with their staff with MS to ensure their needs are being met. In this study, the focus of the MS nurse tended to be around symptom management, aids and adaptations and treatment options rather than other wider but related issues that impact on their health. (e.g. work, relationships, housing and lifestyle). Support from employers was also limited, and none of the participants described an approach that was proactive or ongoing.

The findings from the study support much of the published literature, particularly the study by Jongbloed (1996) which looked at the factors influencing the employment status of women with multiple sclerosis. This identified that women’s decisions about work were usually determined by their social context. Similarly Arnett and Strober (2016) who identified that whilst certain demographics did account for unemployment rates amongst women with MS, social support and also coping style distinguished those who were still employed from those who stopped working due to their MS. However, this study has also shown that type of work, making reasonable adjustments, support from their manager and colleagues and time were key factors. The study also supports the APPG report findings (2017) regarding the need for services to better support people with MS in employment and improve the co-ordination between health, employment and welfare provision.

Changes in UK law and government policy regarding workplace discrimination has meant that women and disabled people now have more rights at work; something that women diagnosed for some time were not able to utilise. However, we know from feminist literature (Lindsey, 2015, Gorp, 2013 and Lewis, 2020) that inequalities in the workplace may pervade due to wider systemic issues that must be tackled to make the workplace more accessible to women. Our research suggests that this may also be the case for disabled people, with most participants reporting negative employment experiences and limited understanding and support from employers. Although it is difficult to disentangle gender and disability effects in the findings from this study participants tended to reflect on their negative employment experiences being due to their disability rather than their gender, potentially due to how the study was framed and interviews structured.

### Strengths and Limitations

The methodology, continuous reflexivity, and a robust and transparent analytical process were all strengths of the study. Another strength was the active involvement of individuals living with MS, which helped to shape the study and aid our interpretation of findings.

The main limitation of the study was the sole use of the MS Society networks to recruit participants, resulting in a sample of women who all had access to support from this organisation. However, a number of network types were used within the MS Society in order to generate a range of experiences (including support groups, fundraising leads and general mailing list). The sample also varied across key characteristics that have previously been shown to influence employment choices and experiences.

### Implications for Policy, Practice and Research

Women with MS that we interviewed were continuing to have negative experiences at work despite national UK policy and legislation that places emphasis on supporting these groups; highlighting that attention may be needed to support these groups and empower women’s work choices. Given the impact of employment as a wider determinant of health (PHE, 2018) and reduced input into national economy, there is a need for joint working by the Department of Health and Social Care and the Department for Work and Pensions to develop policies and procedures that better support people with long term progressive conditions and take account other protected characteristics such as gender.

Social prescribing may offer a mechanism to increase support for women with MS. Defined as ‘*a mechanism for linking patients with non-medical sources of support within the community’* (University College London 2015, p.6), social prescribing aims to improve patients’ quality of life, health and wellbeing through alternative interventions, including those to support employment. In 2018, the Department for Health and Social Care (DHSC) committed to investing £4.5 m in social prescribing projects across England as part of an increased emphasis on multi-agency and holistic approaches to health care outlined in the Five Year Forward View (NHS England, 2016) The recent GP contract reforms also include the funding of 1000 social prescribing link workers in general practice by 2020/21 through the Additional Roles Reimbursement Scheme (British Medical Association, 2020). Research may be beneficial to explore how best to use these roles to support people with MS in employment.

The use of MS nurses has been highlighted as a key source of support for patients, and there may be some benefit of including wider discussions around employment during these interactions. However, the commissioning of MS services varies across England; resulting in an inequity of provision (MS Trust, 2016) and there is a need for better co-ordination of wraparound services to support MS nurse time.

It would be helpful if further research was carried out with a larger sample of women who have a range of conditions to better understand the relationship between the different variables, for example the experiences of those with more progressive and unpredictable conditions (such as MS) compared to those with more consistent and stable conditions.

Women’s employment has been negatively impacted by the changing landscape of work brought about by the COVID-19 pandemic. Researchers from University College London (Wielgoszewska et al., 2020) found that women were considerably more likely to have stopped work to focus on home schooling; potentially widening gender inequalities in the workplace. However, COVID-19 has provided an opportunity for more flexible working arrangements and home-based working, which many employers are considering adopting long-term (CIPD, 2021). Our research suggests such changes may benefit women with MS in roles that facilitate home-working.

## Conclusions

This study provides enhanced understanding of the experiences and perspectives of women living with MS in England regarding employment. The personal and social resources of participants influenced their employment experiences and choices, and whilst support from MS nurses and employers was also identified as important, availability varied. Compromise was a common theme in the women’s accounts of their employment, as was the notion of time as employment experiences and MS symptoms continued to change. Providing statutory support for employment, for example through MS nurses or social prescribing, may help to ensure all women with MS have the resources they need to engage in paid work and provide reasonable adjustments needed to maintain employment.

## Data Availability

The anonymised data analysed during the study are available from the corresponding author on reasonable request.

## Appendix 1

**Table 2:** Topic guide

Section	Sub section
1.Basic information	•Demographics – age, where live, ethnic group, nationality •Home life—understanding day to day life, who they live with, any caring responsibilities, what they like to do in their free time
2.MS diagnosis	•MS type and year diagnosed •Experience of diagnosis: Who delivered it and support received? •Was work discussed? If so by who, at what stage and what was it discussed? •Main MS symptoms? •How has diagnosis affected their approach/aspirations to work? •What information and support could be given to women who are diagnosed with MS regarding paid employment?
3. Education and employment	•Describe past employment – what roles, how much they enjoyed them, what their colleagues were like etc •Current/most recent work and day to day tasks •How did they feel about work before and after diagnosis –the challenges (negatives / positives?) •Their experience of barriers and facilitators regarding work with MS? •How do/did they manage with work on a daily basis? •Any future plans regarding work? •Did they continue education after school – what level?
4. Support networks	•Who have they talked to about work? •How other people have helped or not helped them to stay in work / stop work / make decisions about work -Professional – at their work -MS support network – do they know many other people with MS, do they go to any groups, use social media etc.? Do they find these helpful? -Family and friends – are they helpful in terms of their MS? Do they feel they can speak to them openly and honestly?
5. Other information	•Opportunity for them to discuss anything else that’s relevant to their MS and work?

## Appendix 2

**Table 3:** COREQ 32-item checklist

No. Item	Guide questions/description	Reported on page No.
Domain 1: Research team and reflexivity		
*Personal characteristics*		
1.Interviewer/facilitator	Which author/s conducted the interview or focus group?	9
2. Credentials	What were the researcher’s credentials? E.g. PhD, MD	9 and title page
3. Occupation	What was their occupation at the time of the study?	9 and title page
4. Gender	Was the researcher male or female?	9
5. Experience and training	What experience or training did the researcher have?	9
*Relationship with participants*		
6. Relationship established	Was a relationship established prior to study commencement?	8
7. Participant knowledge of the interviewer	What did the participants know about the researcher? e.g. personal goals, reasons for doing the research	7–8
8. Interviewer characteristics	What characteristics were reported about the inter viewer/facilitator? e.g. Bias, assumptions, reasons and interests in the research topic	9–10
Domain 2: Study design		
*Theoretical framework*		
9. Methodological orientation and Theory	What methodological orientation was stated to underpin the study? e.g. grounded theory, discourse analysis, ethnography, phenomenology, content analysis	6–10
*Participant selection*		
10. Sampling	How were participants selected? e.g. purposive, convenience, consecutive, snowball	7
11. Method of approach	How were participants approached? e.g. face-to-face, telephone, mail, email	7–8
12. Sample size	How many participants were in the study?	8
13. Non-participation	How many people refused to participate or dropped out? Reasons?	8
*Setting*		
14. Setting of data collection	Where was the data collected? e.g. home, clinic, workplace	8
15. Presence of non-participants	Was anyone else present besides the participants and researchers?	8
16 Description of sample	What are the important characteristics of the sample? e.g. demographic data, date	11–12
*Data collection*		
17. Interview guide	Were questions, prompts, guides provided by the authors? Was it pilot tested?	8–9
18. Repeat interviews	Were repeat interviews carried out? If yes, how many?	9
19. Audio/visual recording	Did the research use audio or visual recording to collect the data?	9
20. Field notes	Were field notes made during and/or after the interview or focus group?	9
21. Duration	What was the duration of the inter views or focus group?	9
22. Data saturation	Was data saturation discussed?	7
23. Transcripts returned	Were transcripts returned to participants for comment and/or correction?	9
Domain 3: analysis and findings		
*Data analysis*		
24. Number of data coders	How many data coders coded the data?	Appendix Table 4
25. Description of the coding tree	Did authors provide a description of the coding tree?	Appendix 4
26. Derivation of themes	Were themes identified in advance or derived from the data?	Appendix 4
27. Software	What software, if applicable, was used to manage the data?	9
28. Participant checking	Did participants provide feedback on the findings?	9
*Reporting*		
29. Quotations presented	Were participant quotations presented to illustrate the themes/findings? Was each quotation identified? e.g. participant number	12–21
30. Data and findings consistent	Was there consistency between the data presented and the findings?	9–10
31. Clarity of major themes	Were major themes clearly presented in the findings?	12–21
32. Clarity of minor themes	Is there a description of diverse cases or discussion of minor themes?	Appendix 4

Developed from: Tong A, Sainsbury P, Craig J. Consolidated criteria for reporting qualitative research (COREQ): a 32-item checklist for interviews and focus groups. *International Journal for Quality in Health Care*. 2007. Volume 19, Number 6: pp. 349 – 357.

## Appendix 3

**Table 4:** Phases of thematic analysis

Phases of Thematic Analysis Phase	Description of the process
1. Familiarising yourself with your data	The interviews were transcribed, usually straight after the interview or within 24 h to make sure the data was accurately recorded, taking care to add explanatory notes. The transcribing often took some time, so the primary researcher returned to the text at a later stage (usually the following day) to read over it which helped with familiarisation
2. Generating initial codes	Data was uploaded to NVIVO 12 after 6 interviews were done. This with familiarisation before starting to identify meanings in the text and organise into initial codes (80 codes were initially identified). Whilst doing this the primary researcher completed the remaining 8 interviews and uploaded the transcriptions to NVIVO 12. The software was used to manage and code the data, create descriptive categories by grouping similar codes (nodes in NVIVO), and selecting quotations to include in the write-up
3. Searching for themes	Initial codes were grouped by the primary researcher that reflected a common and recurring pattern across the data. The primary researcher then worked with the co-authors to group the coding into 12 initial themes and 71 sub themes
4. Reviewing themes:	Further analysis was done to better summarise the common features across the dataset and begin to organise in to more meaningful concepts. Thematic maps were used to organise the data into 10 themes and 65 sub themes
5. Defining and naming themes	Further analysis to refine the themes was done resulting in 7 themes and 26 sub themes
6. Producing the report	The results chapter of the report was written and discussed with the co-authors. This provided a further opportunity to review the data and thematic analysis and select participant quotes to reflect the core meaning of the theme

## Appendix 4. Coding

### Initial codes (N: 80)

Acceptance & denial

Awareness

Barriers to work

Being kind to yourself

Challenges of working with MS

Challenges of different work types

Changes in MS over time

Changes in support available over time

Changes personally

Changes that were made at work by them and others due to MS

Colleagues reactions & support

Compromising

Concerns over how others view them

Contemplation

Coping mechanisms

Determination

Diagnosis experience and delays

Disclosure at work

Discrimination

Emotions

Enablers to work

Energy to challenge

Experiences of MS team

Experiences of MS team – work advice

Experiences of wider health

Factors affecting decision to work after diagnosis

Family circumstances

Family understanding

Feeling pressured

Friend network

Friendships

Frustration at lack of support available

Frustration at others not understanding

GP input

Guilt

Hobbies & pastimes

Home situation

Hopelessness

How women with MS experience and make sense of paid employment in their daily lives

Importance of performance at work

Lack of control

Management support

Managing symptoms

Misconceptions about MS

Mobility aids

MS networks

Negative work experiences

Outlook on work

Pleasures

Positive outlook

Pressures to work

Pressures with kids

Pro active

Reaction of friends

Reaction of family

Reflection

Reliance on family

Reliance on friends

Resilience

Self-reassurance

Strategies to manage symptoms

Stubbornness

Suggestions about improvements

Support and information needs of women re work

Support but not reliance

Symptoms

Tenacity

Their perception of their value at work

Thinking of others

Treatments and drugs

Values

Views of their MS

Voluntary work

Wider relationships

Work & education experience

Work ethic

Work satisfaction

World outside MS

Worries about how their MS impacts on others

Wraparound support

### Initial themes (12) and sub themes (71)

**Table:**

Barriers to work	Challenges of working with MS: •Challenges of different work types Misconceptions about MS Pressures with kids
Contemplation over decisions	Factors affecting decision to work after diagnosis
Emotions	Frustration at others not understanding Hopelessness Frustration at lack of support available Feeling pressured Guilt Worries about how their MS impacts on others
Enablers to work	Changes that were made at work by them and others due to MS Strategies to manage symptoms Coping mechanisms Pressures to work
Experiences of services	Experiences of MS team: •Work advice Experiences of wider health GP input Wraparound support
How women with MS experience and make sense of paid employment	Experiences of work: •Management support: Awareness •Disclosure at work •Colleagues reactions & support •Compromising •Their perception of their value at work •Concerns over how others view them •Negative work experiences •Discrimination Work satisfaction
MS experience	Symptoms Treatments and drugs Mobility aids Diagnosis experience and delays
Not themes	Actual work & education experience: •Voluntary work Home & family situation: •Family circumstances •Home situation
Suggestions about improvements	Support and information needs of women re work
Support networks	Wider relationships Friendships MS networks Friend network Family support Support but not reliance Reaction of family Reliance on friends Reliance on family
Time	Changes in MS over time Changes in support available over time Being kind to yourself Changes personally Acceptance & denial Reflection
Values and outlook	Work ethic Stubbornness World outside MS Positive outlook Outlook on work Hobbies & pastimes Views of their MS Pleasures Lack of control Thinking of others Resilience: •Tenacity •Determination •Pro active •Energy to challenge

### Reviewed themes (10) and subthemes (65)

**Table:**

Barriers to work	Challenges of working with MS: •Challenges of different work types Pressures with kids Misconceptions about MS
Emotions	Hopelessness Feeling pressured Guilt Frustration: •Lack of understanding of others •Lack of support
Enablers to work	Changes that were made at work by them and others due to MS Strategies to manage symptoms Pressures to work
Experience of work	Experiences of work: •Management support •Disclosure at work •Compromising •Colleagues reactions & support •Negative work experiences •Their perception of their value at work •Concerns over how others view them •Discrimination Work satisfaction
Experiences of MS	Symptoms Diagnosis experience and delays Treatments and drugs Mobility aids
Experiences of services	Experiences of MS team: •Work advice Wraparound support: •Negative experiences of wraparound GP input Experiences of wider health
Suggestions about improvements	
Support networks	MS networks Support but not reliance Family support Friend network Wider relationships Reliance on others: •Family •Friends
Time	Acceptance & denial Changes in MS over time Changes in support available over time Reflection Decisions about the future Being kind to yourself Changes personally
Values & outlook	Resilience: •Tenacity •Determination •Pro active •Energy to challenge Positive outlook Views of their MS Outlook on work: •Want to work •Don't want to work Hobbies & pastimes Work ethic World outside MS Lack of control Thinking of others Negative outlook Stubbornness

## Abbreviations

APPG: All Party Parliamentary Group
HM: Her Majesty’s
MS: Multiple sclerosis
NHS: National Health Service
NW: Not working
PHE: Public Health England
UK: United Kingdom
W: Working
